# Risk Factors, Clinical Characteristics, and Prognosis of Acute Kidney Injury in Hospitalized COVID-19 Patients: A Retrospective Cohort Study

**DOI:** 10.3390/medicines8010004

**Published:** 2021-01-07

**Authors:** Panupong Hansrivijit, Kinjal P. Gadhiya, Mounika Gangireddy, John D. Goldman

**Affiliations:** 1 Department of Internal Medicine, UPMC Pinnacle, Harrisburg, PA 17104, USA; kinjal268@gmail.com (K.P.G.); g.mounikareddy07@gmail.com (M.G.); 2 Department of Infectious Diseases, UPMC Pinnacle, Harrisburg, PA 17104, USA; goldmanjd@upmc.edu

**Keywords:** COVID-19, coronavirus, SARS-CoV-2, acute kidney injury, risk factors

## Abstract

**Background:** Acute kidney injury (AKI) is a serious complication of COVID-19. **Methods:** Records of hospitalized adult patients with confirmed SARS-CoV-2 infection from 1 March to 31 May 2020 were retrospectively reviewed. **Results:** Of 283 patients, AKI occurred in 40.6%. From multivariate analyses, the risk factors of AKI in COVID-19 can be divided into: (1) demographics/co-morbidities (male, increasing age, diabetes, chronic kidney disease); (2) other organ involvements (transaminitis, elevated troponin I, ST segment/T wave change on electrocardiography); (3) elevated biomarkers (ferritin, lactate dehydrogenase); (4) possible bacterial co-infection (leukocytosis, elevated procalcitonin); (5) need for advanced oxygen delivery (non-invasive positive pressure ventilation, mechanical ventilation); and (6) other critical features (ICU admission, need for vasopressors, acute respiratory distress syndrome). Most AKIs were due to pre-renal (70.4%) and intrinsic (34.8%) causes. Renal replacement therapy was more common in intrinsic AKI. Both pre-renal (HR 3.2; 95% CI 1.7–5.9) and intrinsic AKI (HR 7.7; 95% CI 3.6–16.3) were associated with higher mortality. Male, stage 3 AKI, higher baseline and peak serum creatinine and blood urea nitrogen were prevalent in intrinsic AKI. Urine analysis and the fractional excretion of sodium and urea were not helpful in distinguishing intrinsic AKI from other causes. **Conclusions:** AKI is very common in COVID-19 and is associated with higher mortality. Characterization of AKI is warranted due to its diverse nature and clinical outcome.

## 1. Introduction

Acute kidney injury (AKI) is a common complication in medical and surgical patients which carries a significant mortality association. From the Grampian Laboratory Outcomes Morbidity and Mortality Study II (GLOMMS-II), approximately 8–18% of hospitalized patients were diagnosed with AKI [1]. Moreover, the incidence of AKI has increased correspondingly with the severity of illness. For example, a multi-national database indicated that AKI occurred in 22% of mechanically ventilated patients [2]. Most of these patients developed AKI within two days after being admitted to the intensive care unit (ICU) [2].

AKI is a well established risk factor for both in-hospital death as well as long-term mortality [1,3]. By mitigating potential risk factors for AKI, clinicians could indirectly improve patients’ survival and quality of life. However, the knowledge on the pathophysiology and clinical impact of AKI in certain disease entities are limited, especially in emerging infectious diseases, such as coronavirus disease 2019 (COVID-19).

COVID-19 is a respiratory disease caused by Severe Acute Respiratory Syndrome Coronavirus-2 (SARS-CoV-2) which was first identified in Wuhan, China, in December 2019, before rapidly spreading to 216 countries, causing 18,142,718 infections, and 691,013 deaths as of 4 August 2020 [4]. The highest disease burden was seen in the United States, bearing up to 26 and 22% of global confirmed cases and deaths, respectively [4].

A recent meta-analysis has shown that the pooled incidence of AKI in COVID-19 was only 8.4% and its incidence was closely associated with an increased mortality rate [5]. The incidence of AKI, however, could be as high as 20% in a recent cohort from the New York City area [6]. Nevertheless, only a small number of studies to date have identified the risk factors for AKI in COVID-19. Hansrivijit et al. conducted a meta-analysis and described that age, diabetes, hypertension and baseline serum creatinine levels were potentially predictive factors for AKI in patients with COVID-19 [5]. Furthermore, even fewer studies have reported the clinical or urine characteristics of COVID-19 patients who developed AKI. These data are essential in understanding the disease development as well as disease progression.

Hence, in this retrospective cohort study, we reviewed the incidence, risk factors, and clinical characteristics (blood chemistries and urine studies) of hospitalized COVID-19 patients with AKI. The knowledge obtained from this study would provide an insight on the clinical impact of SARS-CoV-2-associated AKI.

## 2. Experimental Section

### 2.1. Study Design

This retrospective cohort study included data from seven hospitals under UPMC Pinnacle network located across the state of Pennsylvania. The protocol of this study (identification number: 20E024) has been approved by the UPMC Pinnacle Ethic Committee and UPMC Pinnacle Institutional Review Board on 14 May 2020. Written informed consent was waived due the retrospective design of the study. Our research was conducted in accordance with the Declaration of Helsinki.

### 2.2. Data Source and Patient Population

Data were collected from electronic medical records registered under UPMC Pinnacle COVID-19 Registry from 1 March to 31 May 2020. The inclusion criteria are adult patients ≥18 years of age who were hospitalized to the UPMC Pinnacle network during 1 March to 31 May 2020 for any principal diagnoses with concomitant confirmed SARS-CoV-2 infection by real-time polymerase chain reaction (RT-PCR) from a nasopharyngeal swab. We excluded dialysis-dependent patients, unconfirmed/presumed SARS-CoV-2 infection, non-hospitalized patients, pediatric population (age <18 years), pregnant women, and patients enrolled in clinical trials. For patients with multiple admissions within the study period, the first admission for COVID-19 was reviewed.

### 2.3. Data Collection

Patient charts were individually reviewed. The following data were collected: demographics, co-morbidities, signs and symptoms, laboratory findings, radiographic findings, treatments/interventions, complications and outcomes. The descriptive details and definition of each variable are summarized in the Supplemental Document 1.

In our study, AKI was defined and classified by the Kidney Diseases Improving Global Outcomes (KDIGO) guidelines [7]. The estimated glomerular filtration rate (eGFR) was calculated using the Chronic Kidney Disease Epidemiology Collaboration (CKD-EPI) equation [8]. Similarly, we defined chronic kidney disease (CKD) using the KDIGO recommendations [9]. The onset of AKI was divided into (1) community-acquired AKI, defined as AKI that occurred prior to or within 48 h of being hospitalized; and (2) hospital-acquired AKI, defined as AKI that occurred after 48 h of hospitalization. The etiology of AKI was classified as pre-renal, cardiorenal [10], hepatorenal [11], intrinsic and post-renal AKI. To differentiate between pre-renal and intrinsic AKI, the diagnostic criteria proposed by Heller et al. was followed [12] (Supplemental Figure S1). Post-renal AKI included any causes of obstructive uropathy with or without hydronephrosis.

### 2.4. Study Outcomes

The primary outcome was the incidence of AKI classified by its onset and etiology. The secondary outcomes included death, the need for renal replacement therapy (RRT) and the length of hospital stay.

### 2.5. Logistic Regression Analysis

Clinical risk factors that were significant from standard analyses were included in univariate binary logistic regression analyses. Odds ratios (OR) were reported along with a 95% confidence interval (CI). Variables that remained statistically significant on univariate analysis were included in the multivariate analysis using the logistic regression method adjusted for other covariates.

### 2.6. Sensitivity Analysis

The goodness-of-fit tests of logistic regression analyses were evaluated by the Hosmer–Lemeshow method. The validity of the significant results from the multivariate analysis were tested using the bootstrap method to estimate the end point with an imputed sample size of 1000.

### 2.7. Mortality Analysis

The association between AKI origin (pre-renal and intrinsic cause) was analyzed in the multivariate analysis using the Cox regression model. The results are presented in hazard ratio (HR) along with 95% confidence interval. Survival analysis comparing patients with intrinsic AKI and other AKI was presented using the Kaplan-Meier curve.

### 2.8. Statistical Analysis

All analyses were conducted using SPSS software version 23.0 (IBM Corp., Armonk, NY, USA). Descriptive analyses were reported in percentage (for categorical data) and as the mean ± standard deviation (S.D.) or median (interquartile range; IQR) (for continuous data). Categorical variables were evaluated using the Pearson’s χ^2^ tests or Fisher-Exact tests while two-sample independent t-tests and analyses of variance (ANOVA) were used for the continuous variables. Fisher-Exact tests were opted for if any cell count was less than ten. A *p*-value less than 0.05 was considered statistically significant.

## 3. Results

### 3.1. Baseline Characteristics, Interventions and Patient Outcomes

**Figure 1:**
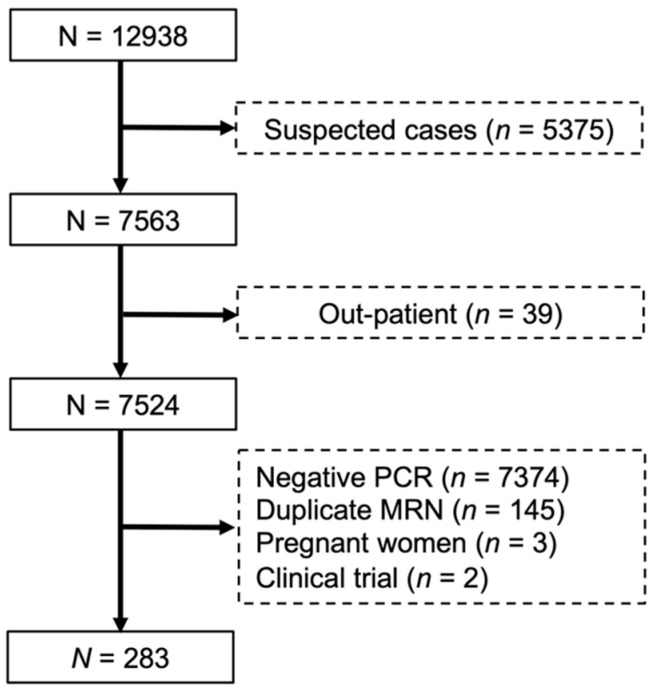
Flowchart of the subject selection from the University of Pittsburgh Medical Center (UPMC) Pinnacle COVID-19 registry from 1 March to 31 May 2020. MRN, medical record number: PCR, polymerase chain reaction.

The patient selection flowchart is illustrated in Figure 1. From a total of 322 patients with confirmed SARS-CoV-2 infection, 39 patients (12.1%) were outpatient and did not require hospitalization resulting in a total of 283 included patients. Table 1 summarizes the demographics and baseline characteristics of included patients. All patients had confirmed SARS-Cov-2 infection by RT-PCR. Of 283 patients, AKI occurred in 40.6% (115/283) of patients. The mean age of all patients was 64.1 ± 15.9 years. Most patients were male (56.2%) and Caucasian (50.5%). Patients who developed AKI were older, male, with a history of hypertension, diabetes mellitus, arrhythmia/conduction disorders, and CKD.

Concerning signs and symptoms, a cough was significantly less frequent while rhonchi was more prevalent in AKI patients.

Laboratory findings showed that leukocytosis, thrombocytopenia, respiratory acidosis, transaminitis, ST segment/T wave changes on electrocardiography (EKG), decreased eGFR on admission, elevated serum creatinine levels, and troponin I levels were common features of patients with AKI. Similarly, the elevation of inflammatory markers, such as D-dimer, ferritin, lactate dehydrogenase (LDH), and procalcitonin were prevalent in patients with AKI.

Patients with AKI likely required non-invasive positive pressure ventilation (NIPPV), mechanical ventilation, intensive care unit admission, vasopressors, hydroxychloroquine, steroids, and tocilizumab.

For complications, arrhythmias, acute respiratory distress syndrome (ARDS), and QT prolongation were more commonly reported in patients who developed AKI. The hospital stay was prolonged, and death was nearly four-fold more prevalent in the AKI group.

### 3.2. Univariate Analysis

All factors except thrombocytosis remained significant in the univariate analysis. The ORs of each clinical predictor for overall in-hospital mortality are depicted in Table 2.

### 3.3. Multivariate Analysis

Variables that were significant on univariate analysis were included in multivariate logistic regression analysis (Table 3). In Model 1 (adjusted for age, sex, ethnicity, and obesity), increasing patient age, male sex, and diabetes were associated with AKI. In Model 2 (adjusted for all variables in Model 1 plus hypertension and diabetes); CKD, leukocytosis, transaminitis, and elevated troponin I levels were associated with AKI. In Model 3 (adjusted for all variables in Model 2 plus CKD); decreasing eGFR, NIPPV, mechanical ventilation, ICU admission, the need for vasopressors, and ARDS were associated with AKI. In Model 4 (adjusted for all variables in Mode 3 plus asthma/COPD and the need for oxygen therapy); respiratory acidosis, elevated ferritin, lactate dehydrogenase, procalcitonin, and steroids use were associated with AKI. Lastly, in Model 5 (adjusted for all variables in Model 3 plus coronary artery disease, heart failure, and history of arrhythmias); only ST-T change on EKG was associated with AKI. All analyses remained significant on bootstrap analysis when the sample size was imputed to 1000.

### 3.4. Characteristics of Patients with AKI Classified by Its Origin

Most AKIs were community-acquired (78.6%) and hospital-acquired (21.4%) (Table 4). The hospital-acquired AKI group had a higher peak serum creatinine, blood urea nitrogen (BUN), and the lowest nadir eGFR. Most stage 1 AKIs were likely community acquired whilst most stage 3 AKIs were hospital-acquired in nature. Both community-acquired AKI and hospital-acquired AKI had a mean fractional excretion of sodium (FE-Na) less than 1.0%. The most common etiology among all causes of AKI was pre-renal (69.4%; 68/98) and intrinsic (25.5%; 25/98). Most community-acquired AKI were due to pre-renal origin while most hospital-acquired AKI were intrinsic. The need for RRT was the highest among hospital-acquired AKI patients. Death was significantly more common in patients with hospital-acquired AKI.

### 3.5. Characteristics of Patients with Intrinsic AKI

Table 5 demonstrates the clinical characteristics of patients with intrinsic AKI. Most patients with intrinsic AKI were male and had higher baseline serum creatinine, peak serum creatinine, and BUN levels compared to other causes of AKI. There was no significant difference in the serum potassium level or urine studies between the two groups. Most patients were in stage 3 AKI for intrinsic AKI while patients with AKI other than intrinsic causes were in stage 1. The fractional excretion of sodium and urea (FE-urea) were similar between the two groups. The need for renal replacement therapy (RRT), length of hospital stay, and death were significantly higher in patients with intrinsic AKI.

### 3.6. Mortality Analysis

**Figure 2:**
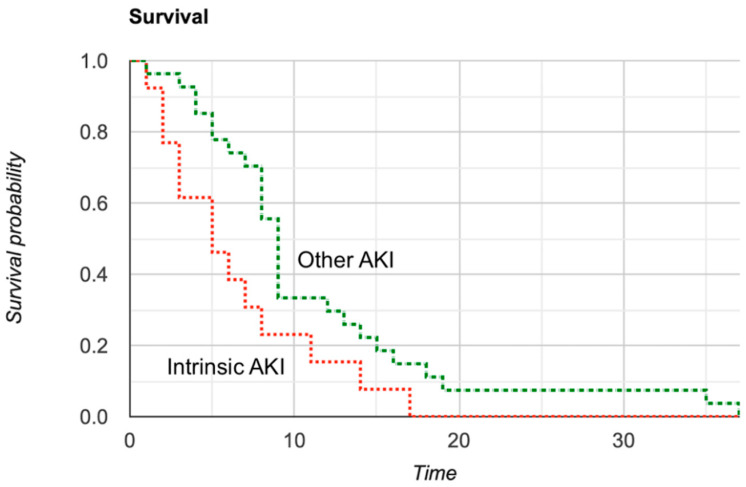
Survival analysis of patients with intrinsic AKI and other AKI using the Kaplan-Meier curve (χ^2^ = 4.04, *p* = 0.04).

We found that the AKI was associated with increased in-hospital mortality (*p* < 0.001) regardless of AKI etiology. From our multivariate analysis, pre-renal AKI was associated with increased overall mortality (HR 3.197; 95% CI 1.725–5.927; *p* < 0.001) after being adjusted for age, sex, ethnicity, obesity, diabetes, hypertension, and chronic kidney disease. Similarly, intrinsic AKI was associated with increased overall mortality (HR 7.701; 95% CI 3.631–16.334; *p* < 0.001) after being adjusted for the same variables. Figure 2 represents the Kaplan–Meier curve analysis comparing patients with intrinsic AKI and other AKI.

## 4. Discussion

In the early stage of the pandemic, one meta-analysis reported the pooled incidence of AKI in COVID-19 at only 8.4% [13]. However, most of their included studies were originated from China and used various diagnostic criteria to define AKI. In our study, the incidence of AKI accounted for up to 40% of patients. This number is almost double compared to the New York City metropolitan area [6] and about eight-fold higher from what was originally reported in China [14]. The data from the United Kingdom also reported 33.4% incidence of AKI with a significant association with death (OR 3.20; 95% CI 2.15–4.81) [15]. In contrast, Lim et al. reported that only 18.3% of hospitalized Korean patients had AKI [16]. By comparing our study, which is considered rural, to the data from the New York City metropolitan area, the discrepancy in the incidence of AKI could be explained by the difference in patient population and racial distribution. Our patients were generally older, Caucasian race, with a higher prevalence of diabetes and CKD. Moreover, residents of nursing homes constituted a significant proportion of our patient population. Not surprisingly, our reported incidence of AKI is similar to a cohort of 13 rural hospitals in the New York Heath System located outside of New York City where most patients reside in long-term living facilities [17]. With this evidence, it is suggested that the incidence of AKI varies by location and region, that is, rural population may have a higher incidence of AKI. Moreover, our study also suggested that most AKIs were community-acquired, and pre-renal in nature, which could be associated with dehydration and poor oral intake, especially in the elderly population. Additional studies comparing the incidence of AKI between the metropolitan area and rural area are needed to confirm our speculation.

The most common etiology among all causes of AKI was pre-renal (70.4%; 81/115) and intrinsic (34.8%; 40/115). Most community-acquired AKIs were due to pre-renal origin while most hospital-acquired AKI were intrinsic. The need for RRT was significantly higher in hospital-acquired and intrinsic AKI. We also confirmed the impact of AKI on the overall mortality, regardless of AKI etiology. Both pre-renal and intrinsic AKI were significantly associated with increased death from COVID-19.

Death was significantly more common in patients with hospital-acquired AKI and in patients with intrinsic AKI. Patients who developed AKI in the hospital tended to be more complicated and critically ill. Multiorgan failure other than the kidneys could also contribute to the higher mortality rate. In addition, we also found that renal replacement therapy was more common in intrinsic AKI. However, we adjusted the association between AKI and death with other co-variates including age, sex, ethnicity, diabetes, hypertension and CKD to minimize confounding bias.

In one meta-regression analysis, increasing age, diabetes, hypertension, and elevated baseline serum creatinine levels were positively associated with AKI [5]. Similarly, the data from South Korea showed that ARDS and low serum albumin on admission were independent risk factors for AKI [16]. Such findings emphasized the importance of defining the risk factors for AKI in these patients in order to prevent death from COVID-19.

We identified several risk factors associated with AKI. In brief, the risk factors of AKI in COVID-19 can be divided into six categories: (1) demographics/co-morbidities (male, age, diabetes, chronic kidney disease); (2) possible liver and cardiac involvement (transaminitis, elevated troponin I, ST-T change on EKG); (3) elevated biomarkers (ferritin, LDH); (4) possible bacterial co-infection (leukocytosis, procalcitonin); (5) the need for advanced oxygen delivery (non-invasive positive pressure ventilation (NIPPV), mechanical ventilation); and (6) other critical features (respiratory acidosis, ICU admission, need for vasopressor, ARDS). Most of these risk factors are consistent with a retrospective cohort from New York Heath System [17] and undoubtedly similar to the data from non-COVID-19 patients [18].

Our findings confirm the risk factors that were identified in previous studies. Age, diabetes, hypertension, and CKD are well known risk factors even in general non-COVID-19 patients. Our findings are also in line with the risk factors reported by the recent meta-analysis [5]. Acute respiratory distress syndrome was also reported as a mutual risk factor in our cohort and in Lim et al. [16]. However, unlike Lim et al., our current study did not examine the effect of serum albumin levels. The significance of hypoalbuminemia toward AKI occurrence has been demonstrated in the literature. A meta-analysis of 168,740 subjects concluded that each 1.0 g/dL decrement of serum albumin levels was associated with a 1.7-fold increased risk of AKI [13]. Although we have identified 17 risk factors for AKI, we believe that there are more potential risk factors for AKI that need to be investigated in the future studies.

The cytokine storm characterized by unregulated proinflammatory cytokine production and excessive cytokine release in response to SARS-CoV-2 infection is a hallmark of clinical deterioration and death from COVID-19 [19,20]. This immunologic response is mediated by several cytokines including IL-1, IL-2, IL-6, IFN-γ. Ferritin, D-dimer and LDH are indirect inflammatory markers and have been recognized as predictive factors for severe COVID-19 and disease progression [21]. With our data, elevated ferritin and LDH could be used as surrogate markers for the risk of AKI as well. Additionally, the presence of transaminitis and elevated troponin I could indicate liver and cardiac involvement resulting from COVID-19 progression, which corresponded with elevated inflammatory biomarkers. In brief, one could conclude that elevated biomarkers caused by COVID-19 are associated with multiple organ involvement which carries a significant mortality. Moreover, elevated procalcitonin and leukocytosis could indicate superimposed bacterial infection which impact the survival from COVID-19. It is speculated that patients with superimposed bacterial infection are more severely ill and would have higher probability of developing AKI.

It is known that the incidence of AKI surges in mechanically ventilated patients. Hirsh et al. described that severe AKI occurred most commonly in close temporal proximity to the time of intubation [17]. Here, we added that advanced oxygen delivery, including NIPPV, was also associated with AKI. Although AKI could result from worsening respiratory failure and distant organ involvement as mentioned above, there is substantial evidence suggesting a direct connection between mechanical ventilation and AKI. Positive pressure ventilation could result in micro-stretching of the alveoli, resulting in proinflammatory cytokine release and the stimulation of renin–angiotensin system that eventually alter the renal perfusion [22,23]. However, more animal studies are encouraged to confirm such findings.

Male, stage 3 AKI, higher baseline serum creatinine, peak serum creatinine and BUN were suggestive of AKI due to intrinsic causes. The pathogenesis of AKI in COVID-19 has not been fully elucidated. Although ischemic acute tubular necrosis (ATN) often occurs in the setting of circulatory collapse and several infections, the systemic prothrombotic state associated with COVID-19 could result in thrombotic microangiopathy of the kidneys. Similar to Hirsch et al. [17], our results showed that urine analysis and urine chemistry, such as FE-sodium and FE-urea, are not helpful in distinguishing between intrinsic AKI from other causes of AKI, as both entities had FE-Na and FE-urea less than 1 and 35%, respectively. Although these findings are traditionally indicative of pre-renal state, glomerulonephritis or certain forms of ATN could give the same results.

The question remains, “*Does SARS-CoV-2 directly involve the kidneys?*” A recent important datum derived from post-mortem kidney biopsies has shown that the most common pathologic diagnosis in patients who died from COVID-19 was acute tubular injury with focal acute tubular necrosis [24,25]. Thrombotic microangiopathy or other glomerular manifestations were not observed. Immunohistochemical assays from SARS-CoV-2 nucleocapsid protein were negative in all patients suggesting no direct evidence of viral infection in the kidneys [24,25]. However, these findings are contradictory to the previous study from China. Su et al. found clusters of coronavirus-like particles in the tubular epithelium and podocytes along with positive immunostaining with SARS-CoV-2 nucleoprotein antibody and suggested that SARS-CoV-2 could directly infect the renal tubular cells [26]. Taken together, although it is possible that the absence of viral particles in the renal tubules from Santoriello et al. [24] and Golmai et al. [25] could be explained by post-mortem RNA degradation, a concept of direct renal tubules infiltration by SARS-CoV-2 needs to be revisited once more data are available.

Our study is among the earliest studies to entail the clinical characteristics and significance of AKI in COVID-19 from a racially diverse population. However, some limitations should be noted. The small sample size of our study remains the major limitation. The hospitalization criteria were not identified specifically for each hospitalized patient. Thus, comparing the difference between hospitalized and non-hospitalized groups might not be practical. Although intrinsic AKI was diagnosed using established criteria, there was, however, no confirmation kidney biopsy. Moreover, the microscopy of the urine was not performed according to the hospital policy to prevent SARS-CoV-2 exposure among technicians. FE-Na, FE-urea, and urine analysis were not available in all patients with AKI which might reduce the robusticity of the analysis. Furthermore, at the beginning of the pandemic, RRT was underperformed at our institution due to bed capacity and staff shortage. As a result, the analysis of predictive factors for RRT cannot be performed due to the small sample size of patients who received RRT.

## 5. Conclusions

AKI is common in COVID-19. Several risk factors of AKI were identified. Most community-acquired AKIs were of pre-renal origin whilst most hospital-acquired AKIs were intrinsic. The need for RRT was significantly more common in intrinsic AKI. Male, higher baseline serum creatinine, peak serum creatinine and BUN, and stage 3 AKI were associated with intrinsic AKI. Both pre-renal and intrinsic AKI were significantly associated with increased mortality. Urine studies and analysis may not be helpful in differentiating intrinsic AKI from other causes of AKI.

## Figures and Tables

**Table 1:** Demographics and baseline characteristics of the included patients.

Characteristics	All Patients (*n* = 283)	No AKI (*n* = 168)	AKI (*n* = 115)	*p*-Value
Male	159 (56.2)	86 (51.2)	73 (63.5)	0.041 *
Age (year)	64.1 (15.9) †	60.8 (16.4) †	68.8 (14.0) †	<0.001 *
***Ethnicity***				
Caucasian	143 (50.5)	85 (50.6)	58 (50.4)	
African-American	88 (31.1)	48 (28.6)	40 (34.8)	
Hispanic/Latino	32 (11.3)	20 (11.9)	12 (10.4)	0.540
Asian	17 (6.0)	13 (7.7)	4 (3.5)	
Others	3 (1.1)	2 (1.2)	1 (0.9)	
***Co-morbidities***				
Obesity (BMI ≥ 30 kg/m^2^)	132 (46.6)	78 (46.4)	54 (47.0)	0.930
Current/former smokers	109 (38.5)	64 (38.1)	45 (39.1)	0.860
Hypertension	189 (66.8)	104 (61.9)	85 (73.9)	0.035 *
Diabetes mellitus	108 (38.2)	52 (31.0)	56 (48.7)	0.003 *
Hyperlipidemia	121 (42.8)	65 (38.7)	56 (48.7)	0.095
Coronary artery disease	50 (17.7)	25 (14.9)	25 (21.7)	0.137
Heart failure/cardiomyopathy	53 (18.7)	27 (16.1)	26 (22.6)	0.166
Arrhythmia/conduction disorders	45 (15.9)	19 (11.3)	26 (22.6)	0.011 *
Chronic kidney disease	66 (23.3)	22 (13.1)	44 (38.3)	<0.001 *
Asthma/COPD	73 (25.8)	40 (23.8)	33 (28.7)	0.357
Cerebrovascular disease	40 (14.1)	20 (11.9)	20 (17.4)	0.193
***Signs and symptoms***				
Cough	185 (65.4)	121 (72.0)	64 (55.7)	0.004 *
Dyspnea	203 (71.7)	127 (75.6)	76 (66.1)	0.081
Hypoxia (SpO2 < 95%)	178 (62.9)	101 (60.1)	77 (67.0)	0.242
Rhinorrhea	29 (10.2)	18 (10.7)	11 (9.6)	0.754
Fever/chills	179 (63.3)	108 (64.3)	71 (61.7)	0.663
Chest pain	35 (12.4)	25 (14.9)	10 (8.7)	0.121
Headache	28 (9.9)	20 (11.9)	8 (7.0)	0.224
Gastrointestinal symptoms	79 (27.9)	49 (29.2)	30 (26.1)	0.571
Asymptomatic	8 (2.8)	4 (2.4)	4 (3.5)	0.719
Rales/crackles	57 (20.1)	32 (19.0)	25 (21.7)	0.579
Rhonchi	57 (20.1)	27 (16.1)	30 (26.1)	0.039 *
Reduced breath sound	63 (22.3)	40 (23.8)	23 (20.0)	0.449
***Laboratory findings***				
Leukopenia (WBC < 4000/uL)	56 (19.8)	37 (22.0)	19 (16.5)	0.254
Leukocytosis (WBC > 10,000/uL)	80 (28.3)	26 (15.5)	54 (47.0)	<0.001 *
Lymphocytopenia (ALC < 1000/uL)	109 (38.7)	31 (18.5)	26 (22.6)	0.392
Thrombocytopenia (<140,000/uL)	57 (20.1)	14 (8.3)	17 (14.8)	0.088
Thrombocytosis (>400,000/uL)	31 (11.0)	54 (32.1)	55 (48.2)	0.006 *
Respiratory acidosis	43 (21.3)	8 (7.3)	35 (38.0)	< 0.001 *
Transaminitis (ALT > 3X UNL)	33 (12.4)	9 (5.8)	24 (21.6)	< 0.001 *
Serum creatinine (mg/dL) on admission	1.06 (0.72) ‡	1.37 (1.95) †	1.92 (1.38)†	0.005 *
eGFR (mL/min/1.73 m^2^) on admission	64.2 (51.0) †	76.5 (35.2) †	46.2 (22.7)†	<0.001 *
Troponin I (>0.03 ng/mL)	91 (39.1)	31 (22.8)	60 (61.9)	<0.001 *
***Inflammatory markers***				
D-dimer (>500 ng/mL)	135 (80.4)	64 (72.7)	71 (88.8)	0.009 *
Ferritin (>336 ng/mL)	109 (65.3)	47 (52.8)	62 (79.5)	<0.001 *
Lactate dehydrogenase (>200 U/L)	108 (73.0)	50 (62.5)	58 (85.3)	0.002 *
C-reactive protein (>1 mg/dL)	152 (87.4)	82 (86.3)	70 (88.6)	0.651
Procalcitonin (>0.25 ng/mL)	73 (47.1)	27 (32.5)	46 (63.9)	<0.001 *
ST-T change on EKG	84 (31.8)	39 (25.0)	45 (41.7)	0.004 *
***Radiographic findings***				
Opacity/infiltrate	209 (73.9)	122 (72.6)	87 (75.7)	0.568
Ground glass appearance	79 (27.9)	42 (25.0)	37 (32.2)	0.186
Pleural effusion	24 (8.5)	11 (6.5)	13 (11.3)	0.158
Pulmonary congestion	30 (10.6)	15 (8.9)	15 (13.0)	0.269
***Oxygen therapy/delivery***				
Nasal cannula	207 (73.1)	121 (72.0)	86 (74.8)	0.607
High-flow nasal cannula	36 (12.7)	18 (10.7)	18 (15.7)	0.221
NIPPV	28 (9.9)	5 (3.0)	23 (20.0)	<0.001 *
Mechanical ventilation	58 (20.5)	11 (6.5)	47 (40.9)	<0.001 *
***Intervention***				
Intensive care unit	89 (31.4)	33 (19.6)	56 (48.7)	<0.001 *
ECMO	2 (0.7)	1 (0.6)	1 (0.9)	1.000
RRT	16 (5.7)	0 (0)	16 (13.9)	<0.001 *
Vasopressor	53 (18.7)	9 (5.4)	44 (38.5)	<0.001 *
Antibiotics	220 (77.7)	131 (78.0)	89 (77.4)	0.908
***Treatment***				
Azithromycin	182 (64.3)	109 (64.9)	73 (63.5)	0.809
Hydroxychloroquine	67 (23.7)	31 (18.5)	36 (31.3)	0.012 *
Steroids	46 (16.3)	16 (9.5)	30 (26.1)	<0.001 *
Ascorbic acid	57 (20.1)	32 (19.0)	25 (21.7)	0.579
Zinc	54 (19.1)	29 (17.3)	25 (21.7)	0.346
Tocilizumab	12 (4.2)	3 (1.8)	9 (7.8)	0.017 *
Convalescent plasma	36 (12.7)	16 (9.5)	20 (17.4)	0.051
Remdesivir	25 (8.8)	17 (10.1)	8 (7.0)	0.401
***Complications***				
ARDS	53 (18.7)	13 (7.7)	40 (34.8)	<0.001 *
Arrhythmias	31 (11.0)	11 (6.5)	20 (17.4)	0.004 *
QT prolongation	25 (8.8)	9 (5.4)	16 (13.9)	0.018 *
Venous thromboembolism	10 (3.5)	4 (2.4)	6 (5.2)	0.326
Arterial thrombosis	1 (0.4)	0 (0)	1 (0.9)	0.406
Cerebrovascular event	3 (1.1)	1 (0.6)	2 (1.7)	0.568
Myocardial infection	5 (1.8)	1 (0.6)	4 (3.5)	0.162
Heart failure	16 (5.7)	9 (5.4)	7 (6.1)	0.799
Superimposed bacteremia	19 (6.7)	7 (4.2)	12 (10.4)	0.052
Hospital stay (day)	6.0 (7.0) ‡	6.1 (6.2) †	10.1 (8.8) †	<0.001 *
***Outcome***				
Recovery/discharge	223 (78.8)	150 (89.3)	73 (63.5)	<0.001 *
Remained hospitalized	2 (0.7)	1 (0.6)	1 (0.9)	1.000
Death	55 (19.4)	15 (8.9)	40 (34.8)	<0.001 *

ALC, absolute lymphocyte count; ALT, alanine transaminase; ARDS, acute respiratory distress syndrome; BMI, body mass index; COPD, chronic obstructive pulmonary disease; ECMO, extra-corporal membrane oxygenation; eGFR, estimated glomerular filtration rate; EKG, electrocardiography; IQR, interquartile range; NIPPV, non-invasive positive pressure ventilation; RRT, renal replacement therapy; SD, standard deviation; SpO2, oxygen saturation; UNL, upper normal limit; WBC, white blood cell. * statistically significant. † mean (standard deviation). ‡ median (IQR).

**Table 2:** Clinical risk factors for acute kidney injury using univariate binary logistic regression analysis.

Characteristics	Odds Ratio	95% CI	*p*-Value
Male	1.657	1.020–2.693	0.041 *
Age (1-year increment)	1.034	1.017–1.051	<0.001 *
Hypertension	1.742	1.036–2.933	0.036 *
Diabetes mellitus	2.119	1.297–3.460	0.003 *
Arrhythmia/conduction disorders	2.294	1.199–4.357	0.012 *
Chronic kidney disease	4.115	2.294–7.407	<0.001 *
Cough	0.487	0.296–0.803	0.005 *
Rhonchi	1.842	1.017–3.311	0.041 *
Leukocytosis (WBC > 10,000/uL)	4.831	2.770–8.403	<0.001 *
Thrombocytosis (>400,000/uL)	1.908	0.900–4.049	0.092
Respiratory acidosis	7.813	3.401–18.182	<0.001 *
Transaminitis (ALT > 3X UNL)	4.505	2.004–10.101	<0.001 *
Serum creatinine (1.0 mg/dL increment)	1.210	1.036–1.413	0.016 *
eGFR (1.0 mL/min/1.73 m^2^ increment)	0.970	0.961–0.978	<0.001 *
Troponin I (>0.03 ng/mL)	5.495	3.096–9.709	<0.001 *
D-dimer (>500 ng/mL)	2.959	1.280–6.849	0.011 *
Ferritin (>336 ng/mL)	3.460	1.739–6.897	<0.001 *
Lactate dehydrogenase (>200 U/L)	3.484	1.548–7.813	0.003 *
Procalcitonin (>0.25 ng/mL)	3.663	1.887–7.143	<0.001 *
ST-T change on EKG	2.141	1.266–3.623	0.005 *
NIPPV	8.130	2.994–22.222	<0.001 *
Mechanical ventilation	9.901	4.831–20.000	<0.001 *
Intensive care unit admission	3.876	2.288–6.579	<0.001 *
Vasopressor	10.989	5.076–23.810	<0.001 *
Hydroxychloroquine	2.012	1.157–3.509	0.013 *
Steroids	3.356	1.730–6.494	<0.001 *
Tocilizumab	4.673	1.236–17.544	0.023 *
ARDS	6.369	3.205–12.658	<0.001 *
Arrhythmias as complication	3.003	1.379–6.536	0.006 *
QT prolongation	2.857	1.215–6.711	0.016 *

ALT, alanine transaminase; ARDS, acute respiratory distress syndrome; CI, confidence interval; eGFR, estimated glomerular filtration rate; EKG, electrocardiography; NIPPV, non-invasive positive pressure ventilation; WBC, white blood cell. *statistically significant.

**Table 3:** Clinical predictors for acute kidney injury using multivariate binary logistic regression analysis.

Characteristics	Statistics		
	Odds Ratio	95% CI	*p*-Value
**Model 1**			
Male	2.285	1.338–3.902	0.002 *
Age (1-year increment)	1.050	1.029–1.071	<0.001 *
Hypertension	1.087	0.604–1.957	0.780
Diabetes mellitus	1.855	1.094–3.145	0.022 *
**Model 2**			
Chronic kidney disease	2.488	1.302–4.762	0.006 *
Cough	0.578	0.328–1.019	0.058
Rhonchi	1.667	0.880–3.155	0.117
Leukocytosis (WBC > 10,000/uL)	4.525	2.494–8.197	<0.001 *
Thrombocytosis (>400,000/uL)	1.961	0.868–4.425	0.105
Transaminitis (ALT > 3X UNL)	4.405	1.770–10.870	0.001 *
Troponin I (>0.03 ng/mL)	4.032	2.179–7.519	<0.001 *
**Model 3**			
Serum creatinine (1.0 mg/dL increment)	1.081	0.933–1.252	0.300
eGFR (1.0 mL/min/1.73 m^2^ decrement)	1.027	1.016–1.037	<0.001 *
NIPPV	6.329	2.179–18.519	0.001 *
Mechanical ventilation	9.434	4.310–20.408	<0.001 *
Intensive care unit admission	3.597	2.000–6.494	<0.001 *
Vasopressor	10.309	4.505–23.810	<0.001 *
ARDS	4.926	2.342–10.309	<0.001 *
**Model 4**			
Respiratory acidosis	7.042	2.710–18.182	<0.001 *
D-dimer (>500 ng/mL)	1.992	0.766–5.181	0.157
Ferritin (>336 ng/mL)	2.710	1.093–6.711	0.031 *
Lactate dehydrogenase (>200 U/L)	3.663	1.376–9.804	0.009 *
Procalcitonin (>0.25 ng/mL)	3.448	1.595–7.463	0.002 *
Hydroxychloroquine	1.689	0.897–3.185	0.104
Steroids	2.591	1.205–5.556	0.015 *
Tocilizumab	4.032	0.986–16.393	0.052
**Model 5**			
Arrhythmia/conduction disorders	1.721	0.779–3.802	0.179
ST-T change on EKG	1.852	1.020–3.356	0.043 *
Arrhythmias as complication	2.217	0.927–5.319	0.074
QT prolongation	2.114	0.821–5.435	0.121

ALT, alanine transaminase; ARDS, acute respiratory distress syndrome; CAD, coronary artery disease; CKD, chronic kidney disease; CI, confidence interval; COPD, chronic obstructive pulmonary disease; eGFR, estimated glomerular filtration rate; EKG, electrocardiography; NIPPV, non-invasive positive pressure ventilation; WBC, white blood cell. * statistically significant. Model 1 is adjusted for age, sex, ethnicity and obesity. Model 2 is adjusted for age, sex, ethnicity, obesity, hypertension, diabetes. Model 3 is adjusted for age, sex, ethnicity, obesity, hypertension, diabetes, and CKD. Model 4 is adjusted for age, sex, ethnicity, obesity, hypertension, diabetes, CKD, asthma/COPD and need for oxygen therapy. Model 5 is adjusted for age, sex, ethnicity, obesity, hypertension, diabetes, CKD, CAD, heart failure and history of arrhythmia/conduction disorder.

**Table 4:** Clinical characteristics of patients with acute kidney injury classified by origin (*n* = 98).

Characteristics	Community-Acquired AKI (*n* = 77)	Hospital-Acquired AKI (*n* = 21)	*p*-Value
Male	48 (62.3)	11 (52.4)	0.456
Age (year) †	67.6 (15.1)	71.3 (10.8)	0.214
**Blood chemistry**			
Baseline serum creatinine (mg/dL) †	0.95 (0.34)	0.95 (0.27)	0.894
Peak serum creatinine (mg/dL) †	2.35 (1.69)	3.45 (1.80)	0.018 *
Lowest eGFR (mL/kg/1.73 m^2^) †	38.6 (21.1)	24.2 (18.2)	0.004 *
Blood urea nitrogen (mg/dL) †	49.0 (33.8)	80.8 (42.4)	0.004 *
Serum sodium (mEq/L) §,†	139 (10)	142 (7)	0.213
Serum potassium (mEq/L) §,†	5.1 (5.0)	5.1 (0.9)	0.929
**AKI staging**			
Stage 1	36 (46.8)	5 (23.8)	
Stage 2	24 (31.2)	5 (23.8)	0.021 *
Stage 3	17 (22.1)	11 (52.4)	
**Urine studies** §			
Urine WBC > 3 or positive leukocyte esterase	23 (40.4)	5 (26.3)	0.272
Urine RBC > 3 or positive blood	37 (64.9)	12 (63.2)	1.000
Urine protein			
Trace	1 (1.8)	0 (0)	
1+	21 (36.8)	4 (21.1)	
2+	16 (28.1)	6 (31.6)	0.661
3+	5 (8.8)	3 (15.8)	
***Urine chemistry*** §			
Fractional excretion of sodium † (%, *n* = 19)	0.6 (0.5)	0.2 (0.1)	0.015 *
Fractional excretion of urea † (%, *n* = 14)	18.4 (9.7)	23.4 (21.1)	0.727
***Etiology***			
Pre-renal	64 (83.1)	4 (19.0)	<0.001 *
Cardiorenal	1 (1.3)	1 (4.8)	0.320
Hepatorenal	0 (0)	0 (0)	-
Intrinsic AKI	9 (11.7)	16 (76.2)	<0.001 *
Post-renal	3 (3.9)	0 (0)	0.358
***Co-morbidities***			
Obesity (BMI ≥ 30 kg/m^2^)	30 (39.0)	13 (61.9)	0.083
Current/former smokers	30 (39.0)	10 (47.6)	0.617
Hypertension	56 (72.7)	15 (71.4)	1.000
Diabetes mellitus	33 (42.9)	13 (61.9)	0.144
Hyperlipidemia	35 (45.5)	11 (52.4)	0.627
Coronary artery disease	16 (20.8)	5 (23.8)	0.764
Heart failure/cardiomyopathy	17 (22.1)	5 (23.8)	0.866
Arrhythmia/conduction disorders	16 (20.8)	3 (14.3)	0.505
Chronic kidney disease	25 (32.5)	7 (33.3)	0.941
Asthma/COPD	25 (32.5)	5 (23.8)	0.445
Cerebrovascular disease	15 (19.5)	4 (19.0)	0965
***Outcome***			
Need for RRT	4 (5.2)	7 (33.3)	<0.001 *
Hospital stay † (day)	8.4 (7.5)	12.4 (9.9)	0.098
Death	16 (20.8)	14 (66.7)	<0.001 *

AKI, acute kidney injury; BMI, body mass index; COPD, chronic obstructive pulmonary disease; eGFR, estimated glomerular filtration rate; RBC, red blood cell; RRT, renal replacement therapy; WBC, white blood cell. * statistically significant. † mean (standard deviation). § at time of AKI.

**Table 5:** Clinical characteristics of patients with intrinsic acute kidney injury.

Characteristics	Intrinsic AKI (*n* = 40)	Other Causes of AKI (*n* = 75)	*p*-Value
Male	31 (77.5)	42 (56.0)	0.026 *
Age (year) †	69.8 (11.1)	68.2 (15.4)	0.551
Hospital-acquired	16 (40.0)	5 (6.7)	<0.001 *
**Blood chemistry**			
Baseline serum creatinine (mg/dL) †	1.20 (0.40)	0.90 (0.32)	<0.001 *
Peak serum creatinine (mg/dL) †	4.35 (1.95)	1.93 (1.13)	<0.001 *
Lowest eGFR (mL/kg/1.73 m^2^) †	17.8 (13.1)	41.8 (19.7)	<0.001 *
Blood urea nitrogen (mg/dL) †	85 (36)	45 (30)	<0.001 *
Serum sodium (mEq/L) §,†	142 (8)	140 (10)	0.172
Serum potassium (mEq/L) §,†	5.2 (0.8)	5.0 (5.1)	0.742
**AKI staging**			
Stage 1	5 (12.5)	41 (54.7)	
Stage 2	12 (30.0)	22 (29.3)	<0.001 *
Stage 3	23 (57.5)	12 (16.0)	
**Urine studies** §			
Urine WBC or leukocyte esterase	15 (40.5)	18 (32.7)	0.444
Urine RBC or blood	27 (73.0)	32 (58.2)	0.147
Urine protein			
Trace	2 (5.4)	2 (3.6)	
1+	9 (24.3)	22 (40.0)	
2+	10 (27.0)	14 (25.5)	0.149
3+	8 (21.6)	3 (5.5)	
**Urine chemistry** §			
Fractional excretion of sodium † (%, *n* = 19)	0.7 (0.6)	0.4 (0.2)	0.084
Fractional excretion of urea † (%, *n* = 14)	23.8 (13.2)	20.1 (13.4)	0.616
**Outcome**			
Need for RRT	16 (40.0)	0 (0)	<0.001 *
Hospital stay † (day)	12.6 (9.4)	8.7 (8.1)	0.030 *
Death	27 (67.5)	13 (17.3)	<0.001 *

AKI, acute kidney injury; eGFR, estimated glomerular filtration rate; RBC, red blood cell; RRT, renal replacement therapy; WBC, white blood cell. * statistically significant. † mean (standard deviation). § at time of AKI.

## Data Availability

No new data were created or analyzed in this study. Data sharing is not applicable to this article.

## Supplementary Materials

The following are available online at https://www.mdpi.com/2305-6320/8/1/4/s1, Supplemental Figure S1. Classification criteria of pre-renal acute kidney injury and intrinsic acute kidney injury. Supplemental Document 1. Detailed explanation of the data collection process.
