# Supplementary material for: Risk Factors, Clinical Characteristics, and Prognosis of Acute Kidney Injury in Hospitalized COVID-19 Patients: A Retrospective Cohort Study
Source: Medicines (Basel). 2021 Jan 7;8(1):4. doi: 10.3390/medicines8010004 (PMC7825666; doi:10.3390/medicines8010004)
Supplement: Supplementary file 1 [file medicines-08-00004-s001.pdf]

Supplementary Materials: Risk Factors, Clinical Characteristics, and Prognosis of Acute Kidney Injury in Hospitalized COVID-19 Patients: A Retrospective Cohort Study

Panupong Hansrivijit, Kinjal P. Gadhiya, Mounika Gangireddy and John D. Goldman

1. Data collection (con't):

The demographic data included age, sex, and ethnicity. Co-morbidities included current/prior history of smoking, obesity (BMI ≥ 30 kg/m^2), hypertension, diabetes mellitus, hyperlipidemia, coronary artery disease, heart failure/cardiomyopathy, arrhythmia, chronic kidney disease, end-stage kidney disease (ESKD), asthma/chronic pulmonary obstructive disease, and cerebrovascular diseases.

Laboratory findings were recorded: leukocytosis (white blood cells count >9500/μL), leukopenia (white blood cells count <3900/μL), lymphocytopenia (absolute lymphocytes count <600/μL), thrombocytosis (platelets >400,000/μL), and thrombocytopenia (platelets <140,000/μL), respiratory acidosis (arterial pH < 7.35 and arterial partial pressure of CO_2 >45 mmHg), transaminitis (alanine transaminase >3 times of upper normal limit). Serum creatinine (mg/dL) was measured by enzymatic assay. Elevation of serum D-dimer (>500 ng/mL), ferritin (>336 ng/mL) lactate dehydrogenase (LDH; >200 U/L), C-reactive protein (>1 mg/dL), procalcitonin (>0.25 ng/mL), and troponin I (>0.03 ng/mL) were recorded. Acute respiratory distress syndrome (ARDS) was defined by the Berlin criteria [16]. Arrhythmias as complications were defined either brady- or tachyarrhythmia that were new-onset, occurred during hospitalization with COVID-19. In this study, Prolongation of QT segment was defined as the QT duration >500 milliseconds.

Clinical Criteria	Prerenal AKI	Intrinsic AKI
Major criterion		
Response to volume repletion	Rapid decrease of creatinine with convergence to baseline levels	No reconstitution of renal function
Minor criteria		
History	Dehydration, loss of extracellular fluid, heart failure, liver failure, inadequate use of diuretics, etc.	Prolonged shock, exposition to nephrotoxins, extrarenal suggestive symptoms e.g. pulmonary syndrome, etc.
Physical examination	Low blood pressure, low jugular pulse, tachycardia, orthostatic changes, poor skin turgor	Absence of signs of dehydration, cardiac monitoring shows adequate volemia
Urinary examination	Absence of proteinuria, hematuria, and leukocyturia	Proteinuria and/or hematuria and/or leukocyturia

AKI, acute kidney injury

The diagnosis of prerenal AKI and intrinsic AKI requires one major criterion AND two minor criteria

Adapted from Heller F, Frischmann S, Grunbaum M, et al. Urinary calprotectin and the distinction between prerenal and intrinsic acute kidney injury. Clin J Am Soc Nephrol 2011;6(10):2347-2355.

Figure S1. Classification criteria of pre-renal acute kidney injury and intrinsic acute kidney injury. Supplemental Document 1. Detailed explanation of the data collection process.
